# Mitochondrial fusion induced by Mdivi-1 and leflunomide alters cell proliferation and viability, steroidogenesis and oxidative stress in chicken ovarian cells

**DOI:** 10.1016/j.psj.2026.106850

**Published:** 2026-03-21

**Authors:** Noemie Couty, Christelle Rame, Lucille Berthet, Claire Chevaleyre, Christine Péchoux, Pascal Froment, Joelle Dupont

**Affiliations:** a CNRS, INRAE, Université de Tours, PRC, F-37380, Nouzilly, France; b Université Paris-Saclay, INRAE, AgroParisTech, GABI, 78350, Jouy-en-Josas, France

**Keywords:** Mitochondria, Fusion, Granulosa, Theca, Hen

## Abstract

Mitochondria are key organelles that regulate energy production, oxidative stress, and steroidogenesis. They are highly dynamic, continuously undergoing fusion and fission. While disruptions in these processes affect follicular development and fertility in mammals, little is known about their role in avian species. This study investigated the expression and functional roles of mitochondrial fusion in hen granulosa and theca cells isolated from dominant (F1) and smaller (F3/F4) follicles. Gene expression analysis revealed that fusion-related genes (*MFN1, MFN2*, and *OMA1*) are more highly expressed in theca cells than in granulosa cells. Furthermore, *MFN1* and *MFN2* levels significantly increase as follicles mature from the F3/F4 to the F1 stage, suggesting that mitochondrial fusion is closely linked to follicular development. To explore the functional impact, cells were treated with Mdivi-1 and leflunomide; both compounds successfully promoted mitochondrial fusion, as evidenced by elongated mitochondrial morphologies. However, their effects on mitochondrial homeostasis differed: leflunomide, but not Mdivi-1, significantly increased fusion gene expression and reduced mitochondrial DNA (mtDNA) copy number (*ND4, ND6, ATP6*). Inducing mitochondrial hyperfusion generally impaired cell viability and proliferation. High doses of both inhibitors suppressed proliferation across both cell types, molecularly confirmed by the downregulation of the pro-proliferative gene *CCND1* and the upregulation of the cell cycle inhibitor *P21*. Regarding steroidogenesis, mitochondrial fusion exerted cell-specific effects. In granulosa cells, both treatments enhanced progesterone secretion, supported by increased expression of *STAR, 3BHSD*, and *CYP11A1*. Conversely, in theca cells, fusion led to decreased testosterone secretion and reduced expression of *CYP11A1* and *CYP19A1*. Finally, while Mdivi-1 did not alter reactive oxygen species (ROS) or ATP levels, leflunomide significantly reduced oxidative stress and increased ATP production. In conclusion, mitochondrial dynamics play a critical, cell-specific role in regulating hen ovarian physiology, influencing the balance between proliferation, metabolism, and steroidogenesis.

## Introduction

Mitochondria are essential regulators of cellular energy metabolism. They are highly dynamic organelles that continuously undergo fusion and fission processes, collectively referred to as mitochondrial dynamics. These processes are critical for maintaining mitochondrial function and cellular homeostasis (Adebayo et al., 2021). In particular, mitochondrial fusion enables the dilution of damage by mixing mitochondrial contents (mitochondrial DNA, proteins, and metabolites), thereby maintaining an efficient respiratory chain and optimizing ATP production. It also limits oxidative stress by redistributing damaged components and supporting the cell’s antioxidant capacity (Youle and Van Der Bliek, 2012). Conversely, mitochondrial fission is essential for cell division, ensuring the proper distribution of mitochondria during mitosis. It also plays a key role in quality control by isolating dysfunctional mitochondria—facilitating their removal through mitophagy—and enabling rapid adaptation to cellular energy demands, notably by modulating ATP production and the generation of reactive oxygen species (ROS). Together, these processes maintain a dynamic balance essential for energy metabolism and the cellular stress response (Twig et al., 2008). In mammals, disruptions in these processes impair mitochondrial integrity, leading to altered cellular metabolism and compromised reproductive competence (Sreerangaraja Urs et al., 2020). Specifically, mitochondria divide into two separate organelles through fission, a mechanism counteracted by fusion, where two mitochondria merge to form a larger one. These dynamics shape the mitochondrial network and determine its overall function. The mitochondrial fusion process is primarily mediated by mitofusin-1 (MFN1) and mitofusin-2 (MFN2) proteins located on the outer mitochondrial membrane, and by optic atrophy 1 (OPA1) and OMA1 (overlapping with m-AAA protease) on the inner membrane (Yu et al., 2020). These proteins act in a coordinated manner to maintain a functional network (Song et al., 2009). In rodents, both MFN1 and MFN2 are required for steroid hormone synthesis, playing conserved roles in cholesterol transport, cholesterol ester storage, and steroidogenesis (Duarte et al., 2012). In contrast, mitochondrial fission is mainly regulated by dynamin-related protein 1 (DRP1), a cytoplasmic GTPase recruited to the mitochondria following phosphorylation (Taguchi et al., 2007). The activity of DRP1 is differentially modulated by phosphorylation at distinct serine residues: for instance, phosphorylation at Ser616 promotes fission by enhancing DRP1 recruitment, whereas phosphorylation at Ser637 inhibits it (Chang and Blackstone, 2007; Cribbs and Strack, 2007; Han et al., 2020). Therefore, a precise balance between fusion and fission is essential for mitochondrial quality control. Mitochondrial dynamics can be pharmacologically modulated by agents such as Mdivi-1 or leflunomide. Mdivi-1 is a selective inhibitor of DRP1 that blocks its phosphorylation at Ser616, thereby inhibiting fission (Deng et al., 2021; Su et al., 2023). More recently, the DHODH inhibitor leflunomide and its active metabolite, teriflunomide, have been reported to modulate fusion–fission dynamics. By binding to the GDP-binding site of DRP1, these molecules act as putative inhibitors of its activity. Additionally, leflunomide inhibits pyrimidine biosynthesis via DHODH, which has been associated with increased expression and activity of MFN1 and MFN2, ultimately promoting mitochondrial fusion (Mirzapoiazova et al., 2024).

Aging hens show a marked decline in egg production after 500 days of age (Abe et al., 1982), accompanied by reduced oocyte quantity and quality (Park et al., 2021). In mammals, alterations in mitochondrial dynamics have been linked to reproductive aging (Wang et al., 2025). Although the precise role of these dynamics in granulosa and theca cells remains poorly explored, disturbances in this balance may alter energy metabolism and steroidogenesis, with potential consequences for follicular development (Chiaratti et al., 2018). In avian species, as in mammals, mitochondria in ovarian somatic cells are central to metabolic activity and steroidogenesis (Diaz et al., 2011). They facilitate the conversion of cholesterol into pregnenolone—the precursor to steroid hormones such as estradiol and progesterone—through pathways dependent on mitochondrial enzymatic activity and cholesterol transport. Furthermore, in mammals, mitochondria supply the ATP required for oocyte maturation, fertilization, and early embryonic development (Wang et al., 2009). Despite their importance, few studies have examined the role of mitochondrial dynamics in hen fertility. To date, only two original studies have reported that an altered fusion/fission balance is associated with follicular atresia and that mitochondrial dysfunction contributes to ovarian aging and reduced steroidogenesis (Hu et al., 2025; Zhan et al., 2024). These findings highlight a clear gap in our understanding of how mitochondrial dynamics directly regulate ovarian physiology and reproductive performance in hens. Within the ovary, mitochondria regulate apoptosis, proliferation, oxidative stress, and steroid production, all of which are essential for follicular function (Chiang et al., 2020). Aging ovarian cells often display mitochondrial hyperfusion, which may represent an adaptive response to sustain energy production but may also disrupt long-term cellular homeostasis (Wang et al., 2025).

Accordingly, the present study aimed to investigate the role of mitochondrial fusion in cellular viability, proliferation, oxidative stress regulation, and progesterone and testosterone secretion in cultured hen granulosa and theca cells isolated from follicles at different developmental stages.

## Materials and methods

### Animal and ethical issues

Thirty-eight White Leghorn laying hens, 35- weeks old, were reared at “Pôle Expérimental Avicole de Tours” (INRAe, Nouzilly, France DOI: 10.15454/1.5572326250887292E12) (PEAT, 2018) under standard conditions, with controlled feeding and a light regime to induce laying. Ovarian follicles were collected at slaughter, by qualified staff. All experiments were approved by the Ethics Committee in Animal Experimentation of Val de Loire CEEA Vdl and registered by the National Committee ‘Comité National de Réflexion Ethique sur l’Expérimentation Animale’ under number 19 (APAFIS #54299-202503130941939 v4). All experiments were performed in accordance with the European Communities Council Directive 2010/63/UE.

### Isolation and culture of chicken granulosa and theca cells

Three independent trials using a total of 38 hens were performed. After electronarcosis, the ovarian follicles were immediately removed and placed in ice-cold sterile 1 % NaCl saline solution. Granulosa cells (GCs) and theca cells (TCs) from preovulatory follicles 1 (F1 = the largest follicle size) and from the 3th and 4th smaller follicles (F3 and F4) were dissected as previously described (Chabrolle et al., 2007; Gilbert et al., 1977; Tosca et al., 2006). We chose to focus on specifically selected F1 and F3/F4 follicles because, as previously shown, granulosa cells from these stages produce differing amounts of progesterone (Tosca et al., 2008). Indeed, under basal conditions (in the absence of FSH or IGF-1 treatment), F3/F4 granulosa cells produced less progesterone than F1 cells. In order to obtain sufficient cells, GCs and TCs form F3 and F4 follicles were pooled into a single group, called GCs F3/4 and TCs F3/4, respectively. Then, cells were isolated with 0.3 % collagenase type A (Roche Diagnostic, Meylan, France) in DMEM medium containing 1 % fetal bovine serum (FBS), at 37°C. Cells were recovered by centrifugation (10 min, 800 g at room temperature), washed with fresh medium and counted. Cells were cultured in DMEM medium supplemented with antibiotics (100U/mL penicillin, 100 mg/l streptomycin), 3 mmol/l l-glutamine and 10 % FBS in a 5 % CO_2_ atmosphere at 37°C. Cells were cultured for 24 h with no treatment. After 24 h, GCs and TCs from F1 and F3/4 follicles were stimulated in fresh culture with Mdivi-1 (0, 12.5, 25 and 50 μM) or Leflunomide (0, 50, 100, 150 μM). The choice of the Mdivi-1 concentrations was determined according to the effects observed in human granulosa cells (He et al., 2025) and those for the Leflunomide was based on the effects observed in different cell types RPMI 8226 Myeloma Cell Line (Adamczuk et al., 2021) or human hepatocellular carcinoma cell line HepG2 (Latchoumycandane et al., 2006).

### Transmission electron microscopy

GCs were seeded into insert and treated for 24 h with Mdivi-1 (25 μM) or Leflunomide (100 μM). Then, GCs were fixed for 24 h at 4°C in 2 % glutaraldehyde in 0.1 M sodium cacodylate buffer (pH 7.2) and placed in 0.2 M sodium cacodylate buffer with 0.4 M sucrose. Post-fixation was performed using 1 % osmium tetroxide containing 1.5 % potassium cyanoferrate, followed by contrast staining with 2 % uranyl acetate in distilled water. The cells were gradually dehydrated through a graded ethanol series (ranging from 30 % to 100 %) and embedded in Epon resin (Delta Microscopie – Labège, France). Ultrathin sections (70 nm) were mounted on 200-mesh copper grids and counterstained with lead citrate before observation under a Hitachi HT7700 electron microscope operated at 80 kV. Analysis was carried out at the MIMA2 platform (MIMA2-UMR 1313 GABI, Electron Microscopy Facility, Jouy-en-Josas, France; https://doi.org/10.15454/1.5572348210007727E12). Microphotographs were captured using an AMT–Hitachi 22 charge-coupled device camera (Elexience, Verrière le Buisson, France).

### Cell viability

Granulosa and theca cells from F1 and F3/4 follicles were treated in 96 wells plates for 48 h with different concentration of Mdivi-1 and Leflunomide as described previously. Cell viability was quantified using a commercial kit Cell Countring Kit-8 (CCK8) assay (Sigma-Aldrich, Saint Quentin Fallavier, France). The assay was performed according to the manufacturer recommendations and the absorbance was measured at 450 nm wavelength using a spectrophotometer.

### Cell proliferation

Granulosa cells and TCs were cultured in 96-well plates and stimulated for 24 h with Mdivi-1 or Leflunomide as described previously. GCs and TCS proliferation was evaluated using a BrdU ELISA assay (Sigma-Aldrich, Saint Quentin Fallavier, France) in accordance with the manufacturer’s instructions. Absorbance was measured using spectrophotometer at 405 and 492 nm wavelength.

### RNA extraction and reverse transcription

RNAs from GCs and TCs were extracted from fresh cells and cultured cells. For fresh cells, GCs and TCs form F1 and F3/4 follicles were obtained from 8 Leghorn hens as described in section 2.1. For cultured cells, GCs and TCs were treated in 6 wells plates with several dilution of Mdivi-1 (0, 12.5, 25 and 50 μM) and Leflunomide (0, 50, 100, 150 μM) for 24 h. Total RNA was extracted from each well using 1 mL of Qiazol RNA Isolation Reagents (Qiagen, Courtaboeuf, France). One hundred and fifty microliters of chloroform (VWR chemicals, France) were added to each lysate, the samples were vortexed for 20 s and centrifuged for 10 min at 17000 g at 4°C. The aqueous phase was recovered and mixed with 600 μl of isopropanol (Carlo Erba Reagent, France) and placed on ice for 10 min before centrifugation for 10 min at 17000 g at 4°C. The supernatant was removed and the pellet was resuspended in 750 μl of 75 % ethanol (Carlo Erba, France) and centrifuged for 5 min at 7500 g at 4°C. Finally, the supernatant was removed and the pellet was resuspended with 15 μl of sterile demineralized water. The DNAse treatment was realised. The quantity and the quality of RNA was determined with a NanoDrop 2000 spectrophotometer (Thermo Scientific, USA). The cDNA was obtained by reverse transcriptase (RT) of 1,5 μg of the total RNA in 20 μl of mix containing RT buffer (2 M), deoxyribonucleotide triphosphate (dATP, dCTP, dGTP, and dTTP, 0.5 mM each), 15 μg/μl of oligodT, 0.125 U of ribonuclease inhibitor, and 0.05 U of Moloney murine leukemia virus reverse transcriptase (MMLV). The mixture was kept for 1 h at 37°C.

### Genomic DNA extraction

Samples were incubated in 200 µL lysis buffer (5 mM EDTA, pH 8.0, 200 mM NaCl, 100 mM Tris, pH 8.0, 0.2 % sodium dodecyl sulfate (SDS) with 0.4 mg proteinase K/1 mL digestion buffer, for 1 h at 55°C under agitation. After centrifugation at 14,000 g for 10 min at room temperature, the supernatant was collected and mixed with an equal volume of isopropanol. Following manual mixing, the samples were centrifuged again at 14,000 g for 10 min. The DNA pellet was washed with 400 µL of 70 % ethanol, agitated, and centrifuged at 8,000 g for 10 min. Finally, the pellet was air-dried and resuspended in 20 µL of sterile water. The quantity and the quality of DNA was determined with a NanoDrop 2000 spectrophotometer (Thermo Scientific, USA).

### Quantitative PCR

Quantitative PCR was performed with 3 μl of cDNA (diluted 5 times) or 3 μl of gDNA (dilutes 10 times) mixed with SYBR Green Supermix 1X reagent (Bio-rad, Marnes la Coquette, France), 250 nM of specific primers (Invitrogen by Life Technologies, Villebon-sur-Yvette, France) given in Table 1 and sterile demineralized water. Samples were set up in duplicate in a 384-well plate and a MyiQ Cycle Device (Bio-Rad, Marnes-la-Coquette, France). After incubation for 2 min at 50°C and a denaturation step of 10 min at 95°C, the samples were subjected to 40 cycles (30 s at 95°C, 30 s at 60°C, 30 s at 72°C) followed by the acquisition of the melting curve. The efficiency (E) of the primers was determined from a serial dilution of cDNA and was calculated as *E* = 10−1/slope value and was between 1.8 and 2.0. For each gene, relative abundance was calculated according to primer efficiency (E) and quantification cycle (Cq), where expression = *E*−Cq. The expression of the target gene was expressed relative to the geometric mean of two specific reference genes (glyceraldehyde-3-phasphatedehydrogenase [*GAPDH*], and Elongation factor 1-alpha [*EEF1A*]). The mean of housekeeping genes has been reported as an accurate normalization factor.

**Table 1:** Primers used for quantitative real-time PCR.

Name	Forward primer	Reverse primer
*MFN1*	CGGTGGTTTTGAGCCCATT	GAAGCCTGGCACCCAAATC
*MFN2*	ATGTGCCTGTGACACGTTCAC	TCGAGTGTCAGGCAGCTTCTT
*OPA1*	CCCAAGCAGGATCCAACAA	AACAACTGCAAAGTAACCCAAAGC
*OMA1*	TCACTATGATTTGGGCCATCTG	GATCCGCTGGCCAACAAC
*ND4*	CCAACCACCAACCTGATAGC	TGTGGGATGGAAGAGTGCC
*ND6*	TAACAACAAACCTCACCCAGCC	GTGTGTCTTTTGCTCGGTTGGA
*ATP6*	ATTCTCAAGCCCCTGCCTAC	TCAGAGTTGGATGGTGGAGAGG
*GCG*	GTGGAGGGCTGATAAAACACAAT	TCCAACTCCTTGACCTCTATCC
*Ch18*	GTCTAAGTACACACGGGCGG	CCTTGGATGTGGTAGCCGTT
*CCND1*	CAGAAGTGCGAAGAGGAAGT	CTGATGGAGTTGTCGGTGTA
*P21*	GAAGAGTTGTCCACGATAAGC	TTCCAGTCCTCCTCAGTCC
*STAR*	TGCCATCTCCTACCAACA	CATCTCCATCTCGCTGAAG
*3BHSD*	AGCTGCTCTGGGAAGTCAAC	TTTGGGCCTGCCACCTCTAT
*CYP11A1*	AGCACTTCAAGGGACTGAGC	ACTTGGTCCCAACTTCCACC
*CYP19A1*	TGTTCCATCACGCTATTT	GATTCTTGTTTGGGCTTC
*EEF1A*	AGCAGACTTTGTGACCTTGCC	TGACATGAGACAGACGGTTGC
*GAPDH*	TGCTGCCCAGAACATCATCC	ATCAGCAGCAGCCTTCACTACC

MFN1 : Mitofusin 1; MFN2 : Mitofusin 2; OPA1 : optic atrophy 1; OMA1 : overlapping with m-AAA protease; ND4 : NADH ubiquinone oxidoreductase subunit 4; ND6 : NADH dehydrogenase subunit 6; ATP6 : ATP Synthase Membrane Subunit 6; GCG : Ch18 : chromosome 18; CCND1 : Cyclin D1; P21 : Cyclin dependent kinase inhibitor P21; STAR : steroidogenic acute regulatory gene; 3BHSD : 3-Beta-hydroxysteroid dehydrogenase; CYP11A1 : cytochrome P450 11A1; CYP19A1 : cytochrome P1911A1; EEF1A : Eukaryotic Elongation factor 1-alpha 1; GAPDH : Glyceraldehyde-3-phosphate dehydrogenase (GAPDH).

### Progesterone and testosterone assays

Progesterone (P4) concentration in GCs and testosterone concentration un TCs from F1 and F3/4 follicles were determined in cultured media after culturing for 24 h in the absence (control group, CTL) or in the presence of different concentration of MDIVI-1 and leflunomide as described previously. The concentration of P4 and testosterone was measured by enzyme-linked immunosorbent assay (ELISA) as previously described (Canepa et al., 2008; Estienne et al., 2020). For all tests, intra- and inter-assay coefficients of variation averaged < 10 %. To account for variations in cell number and protein content, the amount of P4 or testosterone per well was normalized to the total protein content in the same well, measured using the bicinchoninic acid (BCA) protein assay (Interchim, Montluçon, France) according to the manufacturer’s instructions.

### Measurement of reactive oxygen species (ROS) contents

The ROS content in chicken granulosa cells was assessed using the ROS-Glo H₂O₂ assay kit (Promega, Charbonnières-les-Bains, France). Following pharmacological treatments, cells were incubated for 4 h with the H₂O₂ substrate solution. In the presence of H₂O₂, the substrate is converted into a luciferin precursor, generating luminescence measured with a Luminoskan Ascent luminometer (Thermo Fisher, USA). The luminescence signal is proportional to H₂O₂ concentration and ROS activity, as specified by the kit instructions.

### Measurement of ATP production

After pharmacological treatment of granulosa and theca cells, ATP concentrations were measured by using the CellTiter-Glo™ ATP Assay Kit (Promega, France), according to the manufacturer’s instructions.

### Statistical analysis

**Fig. 1:**
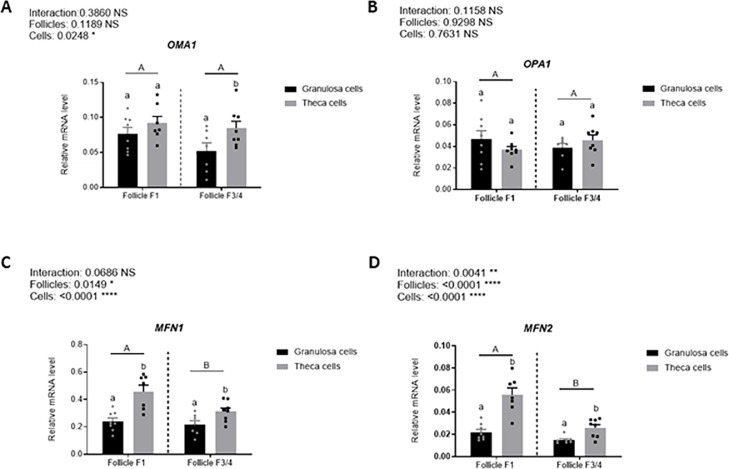
Relative expression of genes involved involved in mitochondrial fusion (*OMA1* (Overlapping with M-AAA protease, Panel A), *OPA1* (OPtic Atrophy-1, Panel B), *MFN1* (MitoFusiN-1, Panel C) and *MFN2* (MitoFusiN-2, Panel D) in granulosa and theca cells follicles at different developmental stages (F1 and F3/4). Values are expressed as means ± SEM, *n* = 8 biological replicates for each condition. Granulosa and theca cells from F1 and F3/4 follicles were sampled from 8 animals. Different capital letters indicate a significant effect of the size of follicle (F1 vs F3/4) whereas lower case letters indicate a significant effect of cell types (granulosa vs theca cells). In each panel, the *P* values are indicated for the follicle type (Follicles, F1 vs F3/4), the cell type (Cells, granulosa cells vs theca cells) and the interaction between these two factors (Interaction). NS: non significant * *P* < 0.05 ** *P* < 0.01 and ****P < 0.0001.

For statistical analysis, we used GraphPad Prism 10 (San Diego, California, USA) with data presented as mean ± standard error of the mean (SEM). The means of independent and random replicates were used, and the Bartlett and Shapiro-Wilk tests were run to test the homogeneity of variance and normal distribution, respectively. Unpaired Student or one-way ANOVA tests followed by a multiple comparisons test were applied for parametric values, and the Mann-Whitney test or the Kruskal-Wallis test followed by Dunn’s multiple comparisons test were applied for non-parametric values. Means were considered different at *P* < 0.05. One-way analysis of variance was used to test differences except for the data reported in Fig. 1 for which a two-way analysis of variance was used. *P* < 0.05 was considered to be statistically significant.

## Results

### Expression of mitochondrial fusion–related genes are more expressed in theca than granulosa cells

We first analysed the expression of genes involved in mitochondrial fusion in granulosa and theca cells, as well as in follicles at different developmental stages. Significant differences were observed between the two ovarian cell types. The expression levels of *MFN1, MFN2*, and *OMA1* were significantly higher in theca cells than in granulosa cells (Fig. 1A, 1C **and** 1D). At the opposite, *OPA1* expression was similar in theca and granulosa cells. We next assessed the effect of follicle size on gene expression. No significant differences in *OPA1* and *OMA1* expression were detected between large (F1) and smaller (F3/4) follicles (Fig. 1A **and** 1B). In contrast, *MFN1* and *MFN2* expression levels were significantly higher in F1 follicles compared with F3/4 follicles (Fig. 1C **and** 1D). A significant interaction between the type of follicle (F1 and F3/4) and the type of ovarian cells (theca an granulosa cells) was detected only for the *MFN2* gene expression.

### Mdivi-1 and leflunomide promote mitochondrial fusion and leflunomide decrease mtDNA copy number in primary chicken granulosa cells

**Fig. 2:**
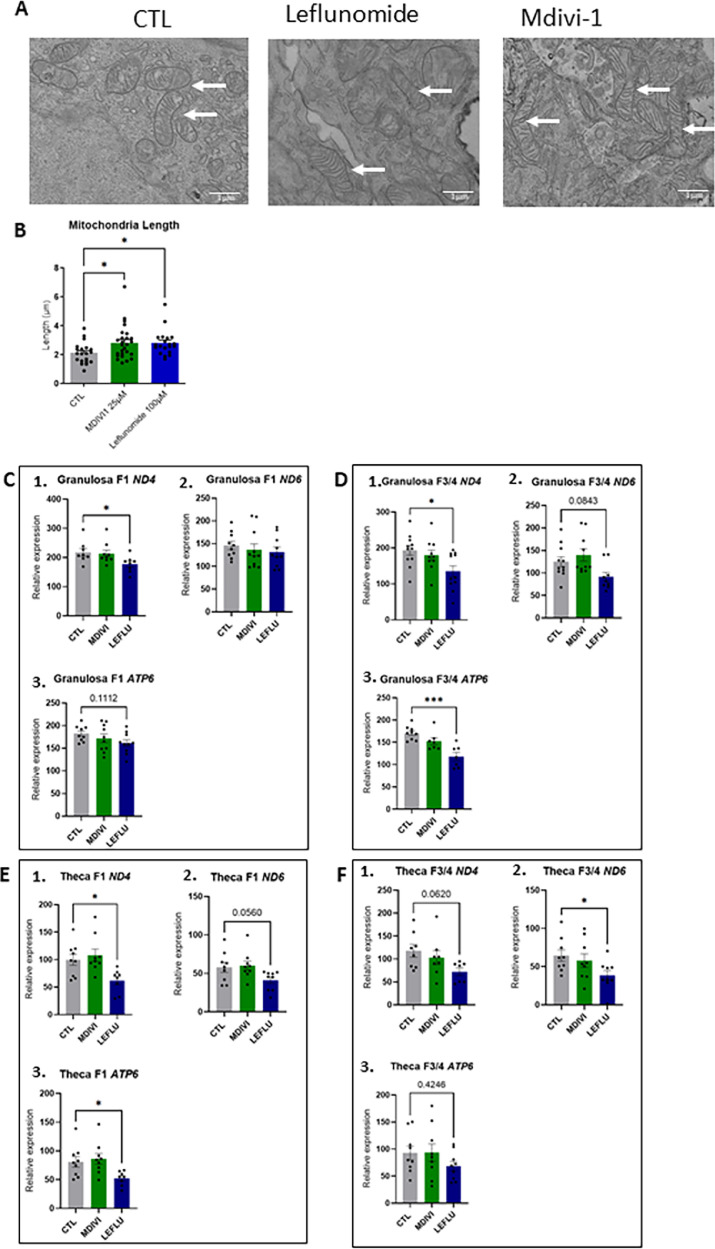
Mitochondria morphology (A and B) in chicken granulosa cells and number of mitochondria in chicken granulosa (C and D) and theca cells (E and F) treated with Leflunomide or Mdivi 1. (A) Transmission electron microscopic micrographs of chicken granulosa cells (*n* = 3) exposed to Leflunomide or Mdivi 1 (*n* = 3). Cells were incubated for 48 h with Leflunomide (25 μM) or Mdivi (100 μM). Scale Bar: 1 μm. Arrows focus on mitochondria. (B) Mitochondria length (µm) was determined by using ImageJ software. Values are means ± SEM. Significant differences are indicated by ***P < 0.0001 and * *P* < 0.05. (C-F) Expression of mitochondrial *ND4, ND6* and *ATP 6* genes in response to Leflunomide (25 μM) and Mdivi (100 μM) in primary chicken granulosa (C and D) and theca cells (E and F) from F1 (C and E) and F3/4 (D and F) follicles. Values are means ± SEM. Significant differences are indicated by *** *P* < 0.0001 and * *P* < 0.05.

Pharmacological treatment with leflunomide resulted in increased expression of mitochondrial fusion–related genes, including *MFN1, MFN2*, and *OPA1* in F1 and F3/4 granulosa cells (Table 2) and in F1 theca cells (Table 3) whereas no significant effect was observed in response to Mdivi-1. These molecular changes observed in response were supported by ultrastructural observations, as electron microscopy revealed elongated mitochondrial morphologies under both treatments (Fig. 2A **and** 2B). Interestingly, leflunomide, but not Mdivi-1, was also associated with a reduction in mitochondrial DNA content, as indicated by decreased expression of *ND4, ND6****,*** and *ATP6* in granulosa (Fig. 2**C and D**) and theca cells (Fig. 2**E and F**) from both F1 and F3/F4 follicles. This finding suggests that the two treatments exert differential effects on mitochondrial homeostasis despite both promoting mitochondrial fusion.

**Table 2:** Relative expression of genes involved in the mitochondrial fusion (*MFN1, MFN2* and *OPA1*) in primary chicken granulosa cells from F1 and F3/4 follicles in response to different concentrations of leflunomide (D1:50 μM; D2: 100 μM et D3: 150 μM) and Mdivi-1 (D1:12.5 μM; D2: 25 μM et D3: 50 μM), two modulators of mitochondrial dynamic.

MOLECULE	Gene	Follicle	CTL	D1	D2	D3	*P* value
***LEFLUNOMIDE***	*MFN1*	**F1**	**0.0009 ±5.485×10^−5 a^**	**0.0013 ± 0.0002^a^**	**0.0015 ± 0.0001^a^**	**0.0020 ± 0.0002^a^**	P = 0.0394 *
		**F3/4**	**0.0011±0.0002^a^**	**0.0017±0.0001^ab^**	**0.0022±0.0001^ab^**	**0.0034 ± 0.0004^b^**	P<0.0001 ****
	*MFN2*	**F1**	**0.0007±8.437×10^−5 a^**	**0.0009±0.000 ^ab^**	**0.0012±8.785×10^−5 ab^**	**0.0014 ± 0.0002^b^**	P = 0.0113 *
		**F3/4**	**0.0008±2.494×10^−5 a^**	**0.0009±7.251×10^−5 ab^**	**0.0013±9.010×10^−5 ab^**	**0.0019 ± 0.0002^b^**	P = 0.0003 ***
	*OPA1*	F1	0.0007 **±** 8.071×10^−5^	0.0011 ± 2.923×10^−5^	0.0012 **±** 8.135×10^−5^	0.0012 **±** 0.0001	*P = 0.0599*
		**F3/4**	**0.0008±0.0002^a^**	**0.0008±8.656×10^−5 a^**	**0.0013±0.0002^a^**	**0.0015 ± 4.394×10^−5 a^**	P = 0.0257 *
***Mdivi-1***	*MFN1*	F1	0.0010 **±** 0.0001	0.0013 ± 0.0002	0.0014 **±** 0.0002	0.0017 **±** 0.0002	*P = 0.1610*
		F3/4	0.0011 **±** 0.0001	0.0012 **±** 0.0002	0.0019 **±** 0.0001	0.0022 **±** 0.0005	*P = 0.0681*
	*MFN2*	F1	0.0014 ± 0.0002	0.0014 ± 0.0003	0.0014 ± 0.0002	0.0017 **±** 0.0003	*P = 0.6709*
		F3/4	0.0010 **±** 5.855×10^−5^	0.0012 ± 0.0002	0.0015 ± 0.0001	0.0018 ± 0.0004	*P = 0.1507*
	*OPA1*	F1	0.0010 ± 0.0002	0.0014 ± 0.0004	0.0012 ± 0.0002	0.0012 ± 0.0003	*P = 0.9905*
		F3/4	0.0007±4.145×10^−5^	0.0011± 0.0002	0.0009±0.0001	0.0012± 0.0002	*P = 0.4090*

Values are expressed as means ± SEM, *n* = 4 independent samples in duplicates at each specific point. One-way ANOVA was performed followed by the Dunn’s test. Letters a and b denotes significant differences at *p* < 0.05. CTL: control (no stimulation with leflunomide or Mdivi-1). *MFN1*: Mitofusin 1; *MFN2*: Mitofusin 2 and *OPA1*: Optic atrophy 1. Mdivi-1 is a mitochondrial division/mitophagy inhibitor and leflunomide is an activator of mitochondrial fusion. Real *P* values are indicated. *P < 0.05; ***P < 0.001; ****P < 0.0001.

**Table 3:** Relative expression of genes involved in the mitochondrial fusion (*MFN1, MFN2* and *OPA1*) in primary chicken theca cells from F1 and F3/4 follicles in response to different concentrations of leflunomide (D1:50 μM; D2: 100 μM et D3: 150 μM) and Mdivi-1 (D1:12.5 μM; D2: 25 μM et D3: 50 μM), two modulators of mitochondrial dynamic.

MOLECULE	Gene	Follicle	CTL	D1	D2	D3	*P* value
**LEFLUNOMIDE**	*MFN1*	F1	0.0013 **±** 0.0003	0.0015 **±** 0.0001	0.0016 ± 0.0002	0.0014 ± 0.0002	*P = 0.6694*
		F3/4	0.0014 **±** 0.0014	0.0018 ± 0.0003	0.0018 ± 0.0003	0.0016±0.0003	*P = 0.8056*
	*MFN2*	**F1**	**0.0005±8.491**×10^−6^**^a^**	**0.0007±5.289×10^−5 ab^**	**0.0008 ±4.812×10^−5 ab^**	**0.0009±6.710×10^−5 b^**	P = 0.0165*
		F3/4	0.0007 **±** 0.0006	0.0009 ± 0.0001	0.0010 ± 0.0002	0.0009 ± 0.0001	*P = 0.1794*
	*OPA1*	**F1**	**0.0005±5.365×10^−5 a^**	**0.0007 ± 3.099**×10^−5^**^a^**	**0.0007 ± 3.791×10^−5 a^**	**0.0008 ± 0.0001^a^**	***P = 0.0515***
		F3/4	0.0007 **±** 0.0001	0.0007±0.0001	0.0008 ± 9.142×10^−5^	0.0008 ± 9.831×10^−5^	*P = 0.9006*
**Mdivi-1**	*MFN1*	F1	0.0015 ± 0.0001	0.0016 ± 0.0002	0.0017± 0.0001	0.0020 ± 0.0003	*P = 0.4373*
		F3/4	0.0022 **±** 0.0008	0.0022 **±** 0.0005	0.0017 **±** 8.419×10^−5^	0.0020 ± 0.0003	*P = 0.9349*
	*MFN2*	F1	0.0008 **±** 9.248×10^−5^	0.0008 **±** 8.391×10^−5^	0.0008 **±** 7.884×10^−5^	0.0011 **±** 8.805×10^−5^	*P = 0.2167*
		F3/4	0.0007±4.706×10^−5^	0.0008 **±** 0.00012	0.0008 **±** 5.434×10^−5^	0.0009 **±** 6.496×10^−5^	*P = 0.2475*
	*OPA1*	F1	0.0007±7.722×10^−5^	0.0008 **±** 0.00022	0.0007 **±** 6.364×10^−5^	0.0009 **±** 4.612×10^−5^	*P = 0.3247*
		F3/4	0.0005 **±** 3.267×10^−5^	0.0007 ± 9.044×10^−5^	0.0007 **±** 4.786×10^−5^	0.0009 **±** 0.0002	*P = 0.1327*

Values are expressed as means ± SEM, *n* = 4 independent samples in duplicates at each specific point. One-way ANOVA was performed followed by the Dunn’s test. Letters a and b denotes significant differences at *p* < 0.05. CTL: control (no stimulation with leflunomide or Mdivi-1). *MFN1*: Mitofusin 1; *MFN2*: Mitofusin 2 and *OPA1*: Optic atrophy 1. Mdivi-1 is a mitochondrial division/mitophagy inhibitor and leflunomide is an activator of mitochondrial fusion. Real *P* values are indicated. *P < 0.05.

#### Mitochondrial fusion reduces granulosa and theca cell viability and proliferation

**Fig. 3:**
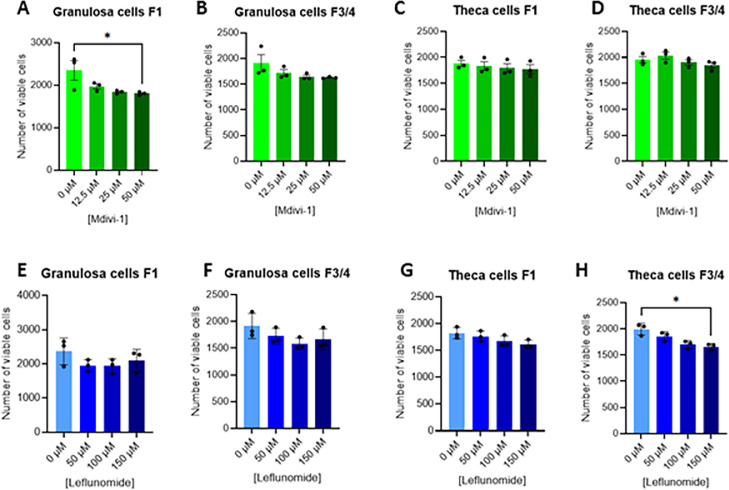
Dose effect of Mdivi-1 (A-D) and Leflunomide (E-H) treatment on primary chicken granulosa (A, B, E and F) and theca (C, D, G and H) cell viability. Primary granulosa and theca cells from F1 and F3/4 follicles were treated with different concentrations of Mdivi-1 (12.5; 25 and 50 μM) and Leflunomide (50; 100 and 150 μM) for 48 h and cell viability was determined using CCK8 assay. Data are means ± SEM of 3 independent experiments. Stars (*) correspond to the one-way ANOVA significance followed the Kruskal Wallis significance followed by the Dunn’s multiple comparison corresponding to the comparison between control (without exposure) and the other Mdivi-1 and Leflunomide concentrations. *: *P*  < 0.05.

**Fig. 4:**
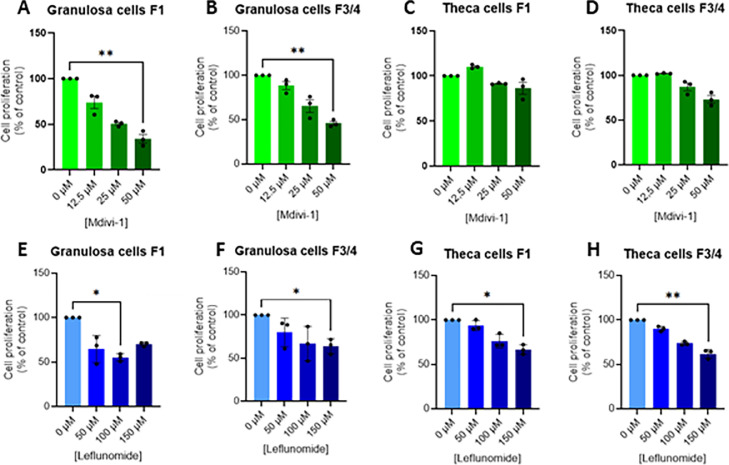
Dose effect of Mdivi-1 (A-D) and Leflunomide (E-H) treatment on primary chicken granulosa (A, B, E and F) and theca (C, D, G and H) cell proliferation. Primary granulosa and theca cells from F1 and F3/4 follicles were treated with different concentrations of Mdivi-1 (12.5; 25 and 50 μM) and Leflunomide (50; 100 and 150 μM) for 24 h and then cell proliferation. Data are means ± SEM of 3 independent experiments. The values were normalized with the control (without no exposure). Stars (*) correspond to the one-way ANOVA significance followed the Kruskal Wallis significance followed by the Dunn’s multiple comparison corresponding to the comparison between control (without exposure) and the other Mdivi-1 and Leflunomide concentrations. *: *P* < 0.05 and **: *P*  < 0.01.

Because the expression levels of some genes involved in mitochondrial dynamics were affected by inhibitor of mitochondrial fusion, we have investigated the functional impact on cell viability (Fig. 3) and proliferation (Fig. 4). Granulosa and theca cells were exposed to Mdivi-1 or leflunomide at increasing concentrations. A significant reduction in cell viability was observed only in F1 granulosa cells treated with Mdivi-1 at 50 µM and in F3/F4 theca cells treated with leflunomide at 150 µM (Fig. 3A). Mdivi-1 treatment significantly reduced proliferation in granulosa cells at 50 µM (Fig. 4A **and** 4B) and in F1 granulosa cells at 100 µM (Fig. 4E). Leflunomide treatment significantly decreased proliferation in F3/F4 granulosa cells (Fig. 4F) and in both F1 and F3/F4 theca cells at 150 µM (Fig. 4G **and** 4H). These inhibitory effects of leflunomide and Mdivi-1 on cell proliferation were confirmed by evaluating *CCND1* and *P21* mRNA expression levels (Tables 4 **and** 5). Indeed, leflunomide treatment (150 μM) inhibited *CCND1* mRNA expression in F3/4 granulosa (Table 4) and theca (Table 5) cells and increased the mRNA expression of the cyclin-dependent kinase inhibitors (CDKIs) P21 in both F1 and F3/4 granulosa (Table 4) and theca (Table 5) cells. In addition, Mdivi-1 treatment (50 μM) increased both *P21* mRNA expression in both F1 and F3/4 theca cells (Table 5) without altering *CCND1* mRNA expression. These results indicate that enhanced mitochondrial fusion negatively affects follicular cell proliferation in a dose- and cell type–dependent manner.

**Table 4:** Relative expression of cell cycle–related genes (*CCND1 and P21*) in primary chicken granulosa cells from F1 and F3/4 follicles in response to different concentrations of leflunomide (D1:50 μM; D2: 100 μM et D3: 150 μM) and Mdivi-1 (D1:12.5 μM; D2: 25 μM et D3: 50 μM), two modulators of mitochondrial dynamic.

MOLECULE	Gene	Follicle	CTL	D1	D2	D3	*P* value
**LEFLUNOMIDE**	*CCND1*	F1	0.0388 ± 0.0036	0.0253 ± 0.0052	0.0253 ± 0.0053	0.0272 ± 0.0056	*P = 0.2916*
		**F3/4**	**0.0483± 0.0022^a^**	**0.0295 ± 0.0027 ^ab^**	**0.0374 ± 0.0019 ^ab^**	**0.0042 ± 0.0026 ^b^**	P = 0.0078 **
	*P21*	**F1**	**0.0027 ± 0.0007^a^**	**0.0037 ± 0.0003 ^ab^**	**0.0053 ± 0.0008 ^ab^**	**0.0067 ± 7.949×10^−5 b^**	P = 0.0048 **
		**F3/4**	**0.0023 ± 0.0005^a^**	**0.0035± 0.0004 ^ab^**	**0.0072 ± 0.0006 ^ab^**	**0.0163 ± 0.0018 ^b^**	P = 0.0003 ***
**Mdivi-1**	*CCND1*	F1	0.0331 ± 0.0025	0.0323 ± 0.0057	0.0253 ± 0.0045	0.0189± 0.0030	*P = 0.1147*
		F3/4	0.0370 ± 0.0127	0.0389 ± 0.0046	0.0305 ± 0.0029	0.0239± 0.0052	*P = 0.4555*
	*P21*	F1	0.0024 ± 0.0004	0.0031 ± 0.0005	0.0034± 0.0006	0.0031 ± 0.0006	*P = 0.7970*
		F3/4	0.0042 ± 0.0022	0.0044 ± 0.0019	0.0038 ± 0.0020	0.0035 ± 0.0013	*P = 0.7297*

Values are expressed as means ± SEM, *n* = 4 independent samples in duplicates at each specific point. One-way ANOVA was performed followed by the Dunn’s test. Letters a and b denotes significant differences at *p* < 0.05. CTL: control (no stimulation with leflunomide or Mdivi-1). *CCND1*: Cyclin D1 and *P21:* Cyclin dependent kinase inhibitor 1. Mdivi-1 is a mitochondrial division/mitophagy inhibitor and leflunomide is an activator of mitochondrial fusion. Real *P* values are indicated. **P < 0.01; ***P < 0.001.

**Table 5:** Relative expression of cell cycle–related genes (*CCND1 and P21*) in primary chicken theca cells from F1 and F3/4 follicles in response to different concentrations of leflunomide (D1:50 μM; D2: 100 μM et D3: 150 μM) and Mdivi-1 (D1:12.5 μM; D2: 25 μM et D3: 50 μM), two modulators of mitochondrial dynamic.

MOLECULE	Gene	Follicle	CTL	D1	D2	D3	*P* value
**LEFLUNOMIDE**	*CCND1*	F1	0.0073 ± 0.0005	0.0071 ± 0.0011	0.0072 ± 0.0005	0.0086 ± 0.0013	*P = 0.8056*
		**F3/4**	**0.0092 ± 0.0004^a^**	**0.0077 ± 0.0002^a^**	**0.0085± 0.0002^a^**	**0.0071 ± 0.0006^a^**	P = 0.0179 *
	*P21*	**F1**	**0.0034 ± 0.0004^a^**	**0.0074 ± 0.0016^a^**	**0.0071± 0.0016^a^**	**0.0108 ± 0.0024^a^**	P = 0.0328 *
		**F3/4**	**0.0024 ± 0.0001^a^**	**0.0052 ± 0.0006^ab^**	**0.0069 ± 0.0002^ab^**	**0.0062 ± 0.0006^b^**	P = 0.0064 **
**Mdivi-1**	*CCND1*	F1	0.0345 ± 0.0091	0.0453 ± 0.0119	0.0364 ± 0.0116	0.0314 ± 0.0050	*P = 0.9006*
		F3/4	0.0345 ± 0.0060	0.0430 ± 0.0059	0.0423± 0.0423	0.0392 ± 0.0046	*P = 0.6098*
	*P21*	**F1**	**0.0064 ± 0.0014^a^**	**0.0167 ± 0.0063^ab^**	**0.0127 ± 0.0036^ab^**	**0.0181 ± 0.0029^b^**	***P = 0.0502***
		**F3/4**	**0.0052 ± 0.0003^a^**	**0.0112 ± 0.0014 ^ab^**	**0.0112 ± 0.0006^ab^**	**0.0123± 0.0013^b^**	P = 0.0382 *

Values are expressed as means ± SEM, *n* = 4 independent samples in duplicates at each specific point. One-way ANOVA was performed followed by the Dunn’s test. Letters a and b denotes significant differences at *p* < 0.05. CTL: control (no stimulation with leflunomide or Mdivi-1). *CCND1*: Cyclin D1 and *P21:* Cyclin dependent kinase inhibitor 1. Mdivi-1 is a mitochondrial division/mitophagy inhibitor and leflunomide is an activator of mitochondrial fusion. Real *P* values are indicated. *P < 0.05; **P < 0.01.

#### Mdivi-1 and leflunomide increase progesterone secretion and decrease testosterone secretion

**Fig. 5:**
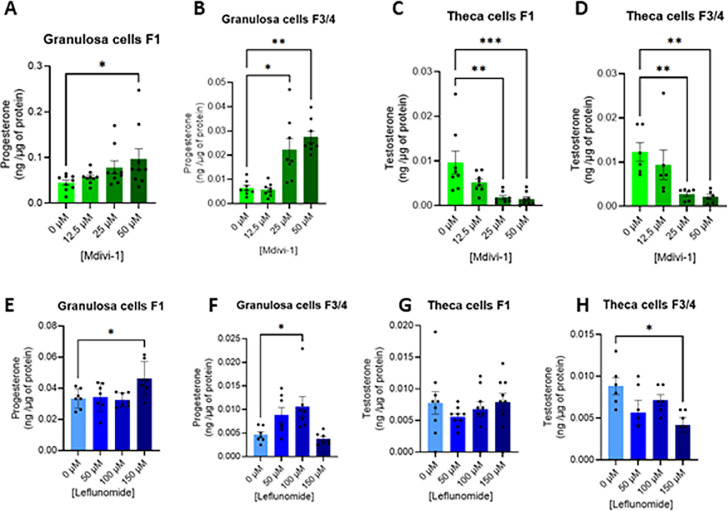
Dose effect of Mdivi-1 (A-D) and Leflunomide (E-H) treatment on progesterone secretion by primary chicken granulosa (A, B, E and F) and on testosterone secretion by theca (C, D, G and H).Primary granulosa and theca cells from F1 and F3/4 follicles were treated with different concentrations of Mdivi-1 (12.5; 25 and 50 μM) and Leflunomide (50; 100 and 150 μM) for 24 h and then progesterone and testosterone were analysed by ELISA assays. Data are means ± SEM of 3 independent experiments. The amount of steroid secreted in ng per well was normalized with the quantity of protein in μg. Stars (*) correspond to the one-way ANOVA significance followed the Kruskal Wallis significance followed by the Dunn’s multiple comparison corresponding to the comparison between control (without exposure) and the other Mdivi-1 and Leflunomide concentrations. *: *P* < 0.05; **: *P* < 0.01 and ***: *P* < 0.001.

Because mitochondrial dynamics are essential for steroidogenic enzyme activity (Duarte et al., 2012), we next evaluated the impact of mitochondrial hyperfusion on steroid hormone secretion (Fig. 5). In presence of elongated mitochondria induced by Mdivi-1 in F1 granulosa cells, a significant increase in progesterone levels was observed at 50 µM concentration (Fig. 5A), whereas in F3/F4 granulosa cells, this effect was already observed at 25 µM (Fig. 5B). Leflunomide also significantly enhanced progesterone secretion, with increases detected at 150 µM in F1 granulosa cells (Fig. 5E) and at 100 µM in F3/F4 granulosa cells (Fig. 5F). These beneficial effects of leflunomide and Mdivi-1 on steroid production were associated with an increase in the mRNA expression of several actors involved in steroidogenesis (Table 6). Specifically, leflunomide treatment (150 µM) increased the mRNA expression of the cholesterol carrier, *STAR* as well as that of the two enzymes *3BHSD* and *CYP11A1* in F1 granulosa cells (Table 6). *CYP11A1* mRNA expression was also increased in F3/4 granulosa cells. Treatment with Mdivi-1 (50 µM) likewise increased *3BHSD* mRNA expression in both F1 and F3/4 granulosa cells and increased *STAR* mRNA expression in F3/4 granulosa cells (Table 6). In contrast, in theca cells, testosterone secretion was reduced following Mdivi-1treatment. Indeed, it significantly decreased testosterone levels at 25 µM and 50 µM (Fig. 5C **and** 5D). Leflunomide induced a significant reduction in testosterone secretion at 150 µM in F3/F4 theca cells (Fig. 5H), whereas no significant effect was observed in F1 theca cells (Fig. 5G). These effects were associated with a decrease in *CYP11A1* mRNA expression in both F1 and F3/4 theca cells, as well as a decrease in *CYP19A1* mRNA expression observed only in F1 theca cells, in response to leflunomide treatment (150 µM) (Table 7). In contrast, Mdivi-1 treatment did not affect the mRNA expression of steroidogenic factors (Table 7). These results indicate that mitochondrial fusion differentially modulates steroidogenesis depending on follicular maturation and cell type.

**Table 6:** Relative expression of steroidogenesis–related genes (*STAR, 3BHSD and CYP11A1*) in primary chicken granulosa cells from F1 and F3/4 follicles in response to different concentrations of leflunomide (D1:50 μM; D2: 100 μM et D3: 150 μM) and Mdivi-1 (D1:12.5 μM; D2: 25 μM et D3: 50 μM), two modulators of mitochondrial dynamic.

MOLECULE	Gene	Follicle	CTL	D1	D2	D3	*P* value
**LEFLUNOMIDE**	*STAR*	**F1**	**0.0156 ± 0.0032 ^a^**	**0.0282 ± 0.0047 ^ab^**	**0.0248 ± 0.0019 ^ab^**	**0.0432± 0.0016 ^b^**	P = 0.0066 **
		F3/4	0.0080 ± 0.0028	0.0076 ± 0.0005	0.0134 ± 0.0059	0.0080 ± 0.0029	*P* = 0.8056
	*3BHSD*	**F1**	**0.1364 ± 0.0122 ^a^**	**0.1930 ± 0.0281 ^ab^**	**0.2210 ± 0.0134 ^ab^**	**0.4293 ± 0.0137 ^b^**	P = 0.0169 *
		F3/4	0.1237 ± 0.0286	0.1355 ± 0.0184	0.1631 ± 0.0208	0.1262 ± 0.0193	*P* = 0.6738
	*CYP11A1*	**F1**	**0.0938 ± 0.0141 ^a^**	**0.1475 ± 0.0115 ^ab^**	**0.1611 ± 0.0113 ^ab^**	**0.1387 ± 0.0143 ^b^**	P = 0.0279 *
		**F3/4**	**0.0700 ± 0.0068 ^a^**	**0.1344 ± 0.0137 ^ab^**	**0.1951 ± 0.0401 ^ab^**	**0.2765 ± 0.0259 ^b^**	*P* = 0.0234 *
**Mdivi-1**	*STAR*	F1	0.0085 ± 0.0043	0.0153 ± 0.0012	0.0176 ± 0.0012	0.0171 ± 0.0040	*P = 0.3216*
		**F3/4**	**0.0059 ± 0.0014 ^a^**	**0.0085 ± 0.0008 ^ab^**	**0.0113 ± 0.0003 ^ab^**	**0.0206 ± 0.0013 ^b^**	*P = 0.0003* ***
	*3BHSD*	**F1**	**0.0880 ± 0.0194 ^a^**	**0.2776 ± 0.0330 ^ab^**	**0.2527 ± 0.0128 ^ab^**	**0.4813 ± 0.0225 ^b^**	***P = 0.0599***
		**F3/4**	**0.0992 ± 0.0062 ^a^**	**0.2225 ± 0.0630 ^ab^**	**0.2055 ± 0.0146 ^ab^**	**0.2708 ± 0.0094 ^b^**	*P =* 0.0205 *
	*CYP11A1*	F1	0.0665 ± 0.0189	0.1261 ± 0,0053	0.1261 ± 0.0042	0.1123 ± 0.0233	*P = 0.0673*
		F3/4	0.0825 ± 0.0057	0.1029 ± 0.0239	0.1020 ± 0.0160	0.1469 ± 0.0195	*P* = 0.2775

Values are expressed as means ± SEM, *n* = 4 independent samples in duplicates at each specific point. One-way ANOVA was performed followed by the Dunn’s test. Letters a and b denotes significant differences at *p* < 0.05. CTL: control (no stimulation with leflunomide or Mdivi-1). *STAR*: steroidogenic acute regulatory protein; *3BHSD* : 3-beta (β)-hydroxysteroid dehydrogenase nd *CYP11A1:* cytochrome P450 family 11 subfamily A member 1. Mdivi-1 is a mitochondrial division/mitophagy inhibitor and leflunomide is an activator of mitochondrial fusion. Real *P* values are indicated. *P < 0.05; **P < 0.01 and ***P < 0.001.

**Table 7:** Relative expression of steroidogenesis–related genes (*STAR, 3BHSD and CYP11A1*) in primary chicken theca cells from F1 and F3/4 follicles in response to different concentrations of leflunomide (D1:50 μM; D2: 100 μM et D3: 150 μM) and Mdivi-1 (D1:12.5 μM; D2: 25 μM et D3: 50 μM), two modulators of mitochondrial dynamic.

MOLECULE	Gene	Follicle	CTL	D1	D2	D3	*P* value
**LEFLUNOMIDE**	*STAR*	F1	9.197×10^−5^ ± 3.838×10^−5^	0.0001 ± 3.594×10^−5^	0.0002 ± 0.0001	0.0001 ± 5.995×10^−5^	*P = 0.5790*
		F3/4	9.613×10^−5^ ± 4.316×10^−5^	0.0001 ± 4.484×10^−5^	0.0002 ± 2.237×10^−5^	0.0002 ± 7.168×10^−5^	*P* = 0.6098
	*3BHSD*	F1	0.0038 ± 0.0009143	0.0060 ± 0.0013	0.0059 ± 0.0011	0.0033 ± 0.0006	*P = 0.2401*
		F3/4	0.0045 ± 0.0008	0.0059 ± 0.0007	0.0067 ± 0.0013	0.0036 ± 0.0007	*P* = 0.2401
	*CYP11A1*	**F1**	**0.005 ± 0.0001 ^a^**	**0.0014 ± 0.0001 ^ab^**	**0.0012 ± 0.0002 ^ab^**	**0.0002 ± 0.0003 ^b^**	P = 0.0069 **
		**F3/4**	**0.007 ± 0.0002 ^a^**	**0.0011 ± 0.0002 ^a^**	**0.0014 ± 0.0003 ^a^**	**0.0003 ± 0.0006 ^a^**	*P* = 0.0299 *
	*CYP19A1*	**F1**	**2.048×10^−5^ ± 6.58×10^−7 a^**	**6.9×10^−5^ ± 2.8** × 10^−6^**^a^**	**3.29×10^−5^ ± 1.23**×10^−6^**^a^**	**1.2** × 10^−7^**± 4.2** × 10^−7^**^b^**	*P* = 0.0284 *
		F3/4	2.572×10^−6^ ± 7.552×10^−7^	5.725×10^−6^ ± 2.566×10^−6^	9.198e-006 ± 3.976×10^−6^	3.976×10^−6^ ± 9.766×10^−7^	*P* = 0.1794
**Mdivi-1**	*STAR*	F1	0.0004 ± 0.0001	0.0003 ± 6.909×10^−5^	0.0003 ± 9.402×10^−5^	0.0001 ± 2.830×10^−5^	*P = 0.1606*
		F3/4	0.0002 ± 8.474×10^−5^	0.0002 ± 8.840×10^−5^	0.0002 ± 7.798×10^−5^	6.5 × 10^−5^ ± 5.3 × 10^−6^	*P = 0.0788*
	*3BHSD*	F1	0.0039 ± 0.0009	0.0047 ± 0.0007	0.0060 ± 0.0012	0.0026 ± 0.0007	*P = 0.2475*
		F3/4	0.0033 ± 0.0007	0.0007 ± 0.0009	0.0069 ± 0.0014	0.0042 ± 0.0008	*P =* 0.3333
	*CYP11A1*	F1	0.0007 ± 0.0003	0.0005 ± 4.980×10^−5^	0.0005 ± 4.073×10^−5^	0.0003 ± 2.572×10^−5^	*P = 0.2775*
		F3/4	0.0005 ± 0.0002	0.0006 ± 9.992×10^−5^	0.0006 ± 9.393×10^−5^	0.0005 ± 0.0001	*P* = 0.9098
	*CYP19A1*	F1	8.117×10^−6^ ± 7.168×10^−6^	4.758×10^−6^ ± 2.209×10^−6^	1.174×10^−5^ ± 5.927×10^−6^	2.397×10^−5^ ± 5.303×10^−6^	*P* = 0.3634
		F3/4	2.407×10^−6^ ± 7.500×10^−7^	7.500×10^−7^ ± 3.749×10^−7^	9.215×10^−6^ ± 4.468×10^−6^	7.953e-006 ± 2.549×10^−6^	*P* = 0.1794

Values are expressed as means ± SEM, *n* = 4 independent samples in duplicates at each specific point. One-way ANOVA was performed followed by the Dunn’s test. Letters a and b denotes significant differences at *p* < 0.05. CTL: control (no stimulation with leflunomide or Mdivi-1). *STAR*: steroidogenic acute regulatory protein; *3BHSD* : 3-beta (β)-hydroxysteroid dehydrogenase nd *CYP11A1:* cytochrome P450 family 11 subfamily A member 1. Mdivi-1 is a mitochondrial division/mitophagy inhibitor and leflunomide is an activator of mitochondrial fusion. Real *P* values are indicated. *P < 0.05 and **P < 0.01.

#### Leflunomide decreases oxidative stress and increases ATP production, whereas Mdivi-1 has no significant effect in granulosa and theca cells

**Fig. 6:**
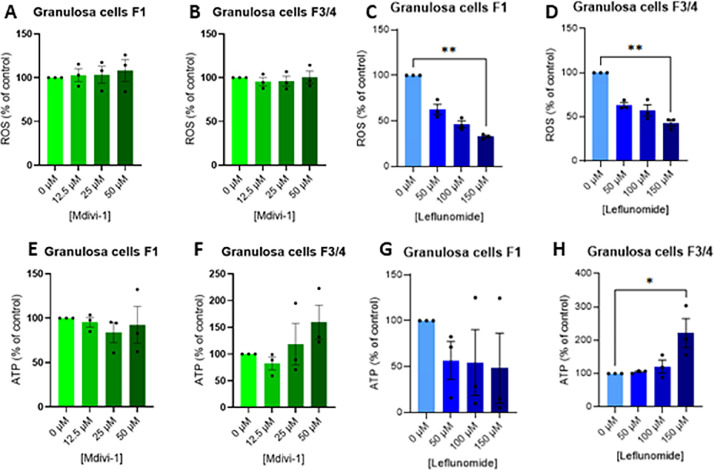
Dose effect of Mdivi-1 and Leflunomide treatment on ROS (A-D) and ATP content (E-H) in primary chicken granulosa cells. Primary granulosa cells from F1 and F3/4 follicles were treated with different concentrations of Mdivi-1 (12.5; 25 and 50 μM) and Leflunomide (50; 100 and 150 μM) for 24 h and then the ROS and ATP contents were analysed as described in the materials and methods. Data are means ± SEM of 3 independent experiments. The values were normalized with the control (without no exposure). Stars (*) correspond to the one-way ANOVA significance followed the Kruskal Wallis significance followed by the Dunn’s multiple comparison corresponding to the comparison between control (without exposure) and the other Mdivi-1 and Leflunomide concentrations. *: *P* < 0.05 and **: *P* < 0.01.

We next examined the effects of induction of mitochondrial fusion on oxidative stress and cellular energy production in granulosa and theca cells (Fig. 6). Mdivi-1 exposition did not significantly modify reactive oxygen species (ROS) content (Fig. 6A **and** B) or ATP production (Fig. 6E **and** F) in granulosa cells from either F1 or F3/F4 follicles, indicating that DRP1 inhibition alone does not alter these parameters under the present experimental conditions. Similar effect was observed in theca cells from either F1 or F3/F4 follicles (data not shown). In contrast, leflunomide treatment induced a dose-dependent decrease in ROS levels in granulosa cells from both follicular stages (Fig. 6C **and** D). Moreover, leflunomide at 150 µM significantly increased ATP production in granulosa cells from F3/F4 follicles, whereas no significant change was observed in F1 granulosa cells (Fig. 6E **and** F). Similar data were obtained in theca cells (data not shown). These findings suggest that leflunomide modulates oxidative stress and mitochondrial energy metabolism in a follicle stage– and dose-dependent manner, while Mdivi-1 exerts minimal effects on these parameters.

## Discussion

**Fig. 7:**
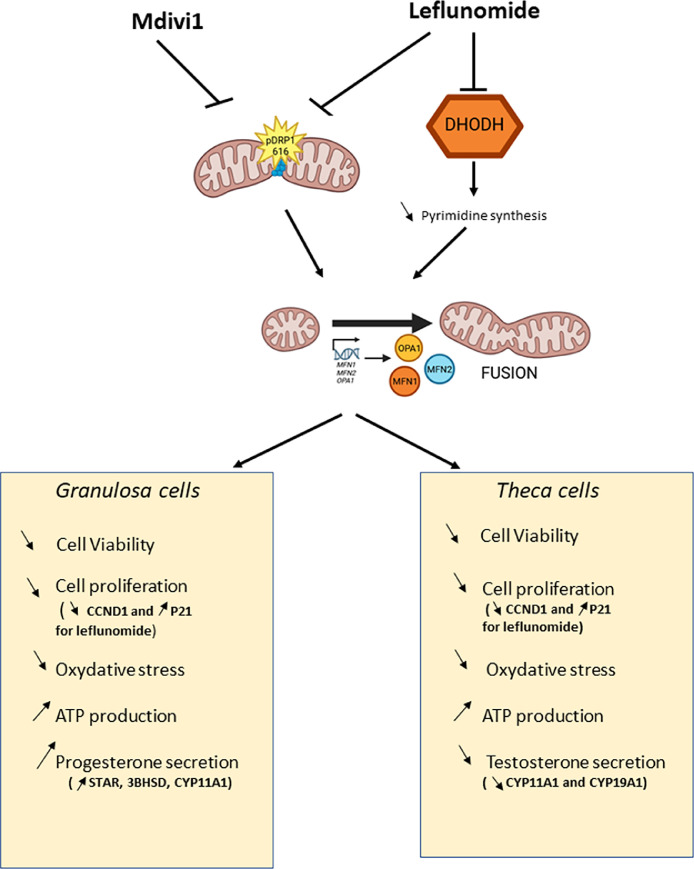
Schematic representation of the Mdivi-1 and leflunomide effects on mitochondrial dynamics and the chicken ovarian cellular functions. Mdivi-1 inhibits DRP1 (Ser616), thereby reducing mitochondrial fission and promoting mitochondrial fusion mediated by OPA1, MFN1, and MFN2. Leflunomide inhibits dihydroorotate dehydrogenase (DHODH), impairing de novo pyrimidine synthesis and influencing mitochondrial function. These alterations modulate the physiology of chicken granulosa and theca cells, leading to changes in cell viability and proliferation—the latter being associated with a downregulation of *CCND1* and an upregulation of the cyclin-dependent kinase inhibitor *P21*. Furthermore, these treatments affect oxidative stress and ATP production. In granulosa cells, progesterone secretion is enhanced, correlated with increased mRNA expression of *STAR, 3BHSD*, and *CYP11A1*. Conversely, in theca cells, testosterone secretion is reduced, associated with a downregulation of *CYP11A1* and *CYP19A1*.

The present study provides novel insights into the role of mitochondrial fusion in avian ovarian cells (granulosa and theca cells) and by pharmacologically modulating mitochondrial dynamics. Our findings demonstrate that mitochondrial fusion–related genes are differentially expressed between ovarian cell types and follicular stages. In addition, we observed that an enhanced mitochondrial fusion significantly impacts cell proliferation and viability, steroidogenesis, oxidative stress, and mitochondrial homeostasis (Fig. 7).

Our results show that genes involved in mitochondrial fusion (*MFN1, MFN2*, and *OMA1*) are expressed at higher levels in theca cells than in granulosa cells, regardless of follicular maturation. This observation suggests more intense mitochondrial activity in theca cells, consistent with their central role in androgen biosynthesis. Indeed, theca cells are the main source of androgen precursors, which are subsequently aromatized by granulosa cells to produce estrogens (Liu et al., 2015). Higher mitofusin expression, may represent a functional adaptation to the high metabolic demands of these cells. Mitofusins regulate not only mitochondrial morphology but also mitochondrial–endoplasmic reticulum contacts, which are essential for lipid and cholesterol trafficking (De Brito and Scorrano, 2008; Duarte et al., 2012). In addition, the higher expression of *OMA1* in theca cells suggests tighter regulation of OPA1 processing and inner membrane remodeling. In mammals, OMA1-mediated cleavage of OPA1 has been implicated in mitochondrial quality control and adaptation to metabolic stress (Baker et al., 2014; Head et al., 2009). Our results therefore extend findings from mammals, suggesting that the metabolic and steroidogenic specialization of theca cells may be partly based on specific regulation of mitochondrial dynamics.

In the present study, we show that *MFN1* and *MFN2* expression increases in F1 follicles compared with F3/F4 follicles, whereas *OPA1* and *OMA1* levels remain stable across follicular stages. This finding suggests that mitofusins, rather than inner membrane remodeling proteins, are selectively upregulated during follicular dominance. Follicle selection and maturation are associated with increased metabolic demand and steroidogenic output (Johnson, 2015). Previous studies in mammals have reported a positive association between mitochondrial biogenesis, fusion, and follicular competence (Van Blerkom, 2011). Moreover, mitochondrial dynamics have been shown to undergo significant changes during follicular growth (Jiang et al., 2021; Udagawa et al., 2014). More recently, MFN2 has been demonstrated to play a crucial role in follicular development and in maintaining the follicular reserve in mice (Zhang et al., 2019).

We confirmed that both Mdivi-1 and leflunomide promote mitochondrial fusion in avian ovarian cells, as evidenced by elongated mitochondrial morphology. These observations are consistent with previous reports in other cell model (Curel et al., 2024; Manczak et al., 2019; Niu et al., 2019; Reddy et al., 2017). However, despite similar effects on mitochondrial morphology, the two compounds exerted distinct effects on mitochondrial DNA content. Leflunomide, but not Mdivi-1, reduced the expression of mtDNA-encoded genes, suggesting impaired mtDNA maintenance or replication. Leflunomide is a known inhibitor of dihydroorotate dehydrogenase (DHODH), a mitochondrial enzyme involved in pyrimidine biosynthesis (Löffler et al., 1997). Inhibition of DHODH can limit nucleotide availability and has been linked to reduced mtDNA replication and altered mitochondrial biogenesis (Mirzapoiazova et al., 2024).

Here, we showed that an enhanced mitochondrial fusion negatively affects cell proliferation and, to a lesser extent, cell viability in a dose-, cell type–, and follicle stage–dependent manner. Our data were confirmed by a reduction in *CCDN1* mRNA expression and an increase in the cyclin dependent kinase inhibitor, *P21* mRNA expression. Our results are in good agreement with previous studies demonstrating that prolonged mitochondrial hyperfusion can lead to cell cycle arrest in cancer cells (Dai et al., 2020; Gong et al., 2025; Mitra et al., 2009; Zhang et al., 2023). In addition, a recent study in chicken demonstrated that inhibition of *MFN2* expression by siRNA in granulosa cells results in decreased levels of PCNA and CDK2 proteins, which play key roles in cell cycle regulation (Hou et al., 2024). Together, these data suggest that a precise regulation of mitochondrial dynamics is required to balance proliferation and differentiation during follicular development.

In the present sudy, we showed that both Mdivi-1 and leflunomide increased progesterone secretion in chicken granulosa cells while reducing testosterone production in theca cells. In addition, in granulosa cells, these changes were associated with the upregulation of the mRNA expression of the cholesterol carrier *STAR* and the steroidogenic enzymes *3BHSD* and *CYP11A1*. Conversely, we observed that the reduced testosterone secretion in theca cells was associated to the downregulation of *CYP11A1* and *CYP19A1* mRNA expression. Our result are in good agreement with a recent paper showing that Mdivi-1 increases progesterone secretion in human granulosa cells (He et al., 2025). Mitochondria are central organelles to steroidogenesis, as the first and rate-limiting step—the conversion of cholesterol to pregnenolone by CYP11A1—occurs in the mitochondrial inner membrane (Chien et al., 2017; Melchinger and Garcia, 2023). In addition, proper mitochondrial morphology and membrane organization are essential for the assembly and activity of steroidogenic enzyme complexes (Rone et al., 2012). Increased mitochondrial fusion may enhance progesterone synthesis by optimizing cholesterol transport and mitochondrial enzyme efficiency in granulosa cells. Conversely, reduced testosterone production in theca cells may reflect altered mitochondrial function or reduced mtDNA-encoded respiratory capacity, particularly following leflunomide treatment. In our study, we also observed that leflunomide, but not Mdivi-1, reduces ROS levels and increases ATP production. This is consistent with reports showing that certain mitochondrial fusion states enhance the efficiency of oxidative phosphorylation (Picard et al., 2015). The difference observed with Mdivi-1 may be attributed to its specific effects on DRP1, which do not necessarily modulate metabolic function but rather mitochondrial morphology (Lackner, 2014). However, it is crucial to note that both leflunomide and Mdivi-1 possess off-target effects that modulate cellular metabolism. Specifically, leflunomide through its inhibition of DHODH directly impacts mitochondrial electron transport and pyrimidine biosynthesis. This can alter ATP production and redox balance independently of its influence on mitochondrial dynamics (Rückemann et al., 1998). Conversely, Mdivi-1 has been shown to inhibit mitochondrial complex I, thereby modulating electron flow and the generation of reactive oxygen species (ROS) independently of DRP1 inhibition (Bordt et al., 2017). Consequently, these off-target effects may contribute to the differential impacts on ROS levels and ATP production observed in our study, and must be considered when interpreting the compounds' effects on mitochondrial function.

Although the present study provides novel insights into the role of mitochondrial fusion in hen ovarian physiology, several limitations should be acknowledged. First, the study was conducted exclusively *in vitro* using isolated granulosa and theca cells. While this approach allows precise mechanistic investigation, it does not fully recapitulate the complex endocrine and paracrine interactions occurring within the intact follicle. Future *in vivo* studies will therefore be required to confirm the physiological relevance of mitochondrial fusion modulation on follicular development, ovulation, and egg production. It appears crucial to determine whether mitochondrial fusion in aged laying hens during egg production is identical to the fusion induced in vitro. Furthermore, a comparison of mitochondrial dynamics within ovarian cells between young and aged hens during the laying period remains to be investigated. If differences in ovarian mitochondrial dynamics are observed between young and aged animals, we could hypothesize that these variations are associated with the decline in laying rate. Consequently, rebalancing mitochondrial dynamics could lead to improved lay persistency, thereby supporting more sustainable egg production. Second, mitochondrial dynamics were mainly assessed through gene expression and ultrastructural observations. Additional functional analyses, such as direct measurements of mitochondrial respiration, membrane potential, and mitophagy, would further strengthen the interpretation of mitochondrial quality control mechanisms. Moreover, although Mdivi-1 and leflunomide are widely used as pharmacological modulators of mitochondrial dynamics, both compounds may exert off-target effects, particularly leflunomide through DHODH inhibition. The use of genetic approaches targeting *DRP1, MFN1, MFN2*, or *OPA1* would help confirm the specificity of the observed effects.

## Conclusion

Taken together, our results demonstrate that mitochondrial fusion is differentially regulated between ovarian cell types and follicular stages and plays a critical role in controlling proliferation, steroidogenesis, oxidative stress, and mitochondrial homeostasis in hen ovarian cells. While promoting mitochondrial fusion enhances progesterone production, excessive or prolonged fusion—particularly when combined with metabolic inhibition—may impair cell proliferation and mitochondrial integrity. These findings underscore the importance of finely tuned mitochondrial dynamics for maintaining ovarian function and suggest that mitochondrial fusion represents a key regulatory mechanism in avian folliculogenesis and reproductive aging. Further exploration of mitochondrial modulators *in vivo* could open new avenues for improving laying performance and reproductive longevity in commercial hens.

## Funding

The authors thank to Région Centre Val de Loire for its support (FERTICHAUD, Grant number 2024 00156299).

## CRediT authorship contribution statement

**Noemie Couty:** Writing – review & editing, Writing – original draft, Validation, Supervision, Methodology, Conceptualization. **Christelle Rame:** Writing – review & editing, Validation, Methodology, Investigation, Formal analysis, Conceptualization. **Lucille Berthet:** Writing – review & editing, Validation, Investigation. **Claire Chevaleyre:** Writing – review & editing, Validation, Investigation. **Christine Péchoux:** Writing – review & editing, Validation, Methodology. **Pascal Froment:** Writing – review & editing, Validation, Methodology, Investigation. **Joelle Dupont:** Writing – review & editing, Writing – original draft, Validation, Supervision, Project administration, Methodology, Investigation, Funding acquisition, Formal analysis, Conceptualization.

## Disclosures

The authors declare that they have no known competing financial interests or personal relationships that could have appeared to influence the work reported in this paper.
